# Immunologic responses to xenogeneic tyrosinase DNA vaccine administered by electroporation in patients with malignant melanoma

**DOI:** 10.1186/2051-1426-1-20

**Published:** 2013-11-18

**Authors:** Jianda Yuan, Geoffrey Y Ku, Matthew Adamow, Zhenyu Mu, Sapna Tandon, Drew Hannaman, Paul Chapman, Gary Schwartz, Richard Carvajal, Katherine S Panageas, Alan N Houghton, Jedd D Wolchok

**Affiliations:** 1 Ludwig Center for Cancer Immunotherapy, Immunology Program, Sloan-Kettering Institute, New York NY10065, USA; 2 Department of Epidemiology and Biostatistics, Memorial Sloan-Kettering Cancer Center, New York NY10065, USA; 3 Ichor Medical System, Inc., San Diego, CA 92121, USA; 4 Department of Medicine, Memorial Sloan Kettering Cancer Center, New York, NY 10065, USA

**Keywords:** Tyrosinase, DNA vaccine, Electroporation, Immune response, Epitope spreading, Melanoma patient

## Abstract

**Background:**

Prior studies show that intramuscular injection and particle-mediated epidermal delivery of xenogeneic melanosomal antigens (tyrosinase or Tyr, gp100) induce CD8^+^ T cell responses to the syngeneic protein. To further define the optimal vaccination strategy, we conducted a phase I study of *in vivo* electroporation (EP) of a murine Tyr DNA vaccine (pINGmuTyr) in malignant melanoma patients.

**Methods:**

Human leukocyte antigen (HLA)-A1, A2, A24 or B35 stage IIb-IV melanoma patients received up to five doses of the mouse tyrosinase DNA vaccine by EP every three weeks at dose levels of 0.2 mg, 0.5 mg, or 1.5 mg per injection. Peripheral blood mononuclear cells (PBMC) were collected, cultured with a peptide pool containing eight HLA class I-restricted Tyr-specific T-cell epitopes, and analyzed by HLA-A*0101-restricted tetramers and intracellular cytokine staining (ICS).

**Results:**

Twenty-four patients received ≥1 dose of the pINGmuTyr vaccine; PBMCs from 21 patients who completed all five doses were available for Tyr immune assays. The only common toxicity was grade 1 injection site reaction. Six of 15 patients (40%) in the 1.5 mg dose cohort developed Tyr-reactive CD8^+^ T cell responses following stimulation, defined as a ≥3 standard deviation increase in baseline reactivity by tetramer or ICS assays. No Tyr-reactive CD8^+^ T cell response was detected in the 0.2 mg and 0.5 mg dose cohort patients. Epitope spreading of CD8^+^ T cell response to NY-ESO-1 was observed in one patient with vitiligo. One patient subsequently received ipilimumab and developed an enhanced Tyr-reactive response with polyfunctional cytokine profile. After a median follow-up of 40.9 months, median survival has not been reached.

**Conclusions:**

A regimen of five immunizations with pINGmuTyr administered by EP was found to be safe and resulted in Tyr-reactive immune responses in six of 15 patients at 1.5 mg dose cohort.

**Trial registration:**

ClinicalTrials.gov NCT00471133

## Background

While most early stage malignant melanomas can be cured by surgical excision alone, the relapse rates of high-risk melanomas (Breslow thickness >4 mm or loco-regional metastases) remain high after surgery [1,2]. The only Food and Drug Administration (FDA)-approved adjuvant therapy for melanoma is high-dose interferon-α (IFN-α), which consistently improves relapse-free survival, but not overall survival, and is associated with significant toxicities [3,4]. Consequently, there is a strong interest in developing more effective and better tolerated adjuvant therapies.

Melanoma is an attractive target for immunotherapy because of its selective expression of differentiation antigens not expressed by other tissues. Strategies to harness the immune system against melanoma have included cytokine therapy, immune-modulating antibodies, adoptive T-cell therapy, and vaccines [5]. These efforts culminated in the milestone approval of ipilimumab, a monoclonal antibody against cytotoxic T-lymphocyte antigen-4 (CTLA-4), in March 2011 for patients with refractory melanoma [6]. In another disease setting, castrate-resistant prostate cancer, the approval of Sipuleucel-T, a dendritic cell vaccine, demonstrated that tumor vaccination strategies have the potential to provide clinical benefit for advanced cancer patients [7].

Many different vaccination approaches have been attempted in melanoma, including whole-cell vaccines, peptide/protein-based vaccines, ganglioside vaccines, dendritic-cell based therapy, recombinant viral vectors and DNA vaccines [5]. DNA vaccines allow direct delivery of antigen into the MHC class I-processing pathway, which is necessary to elicit cytotoxic T-cell responses [8]. Other advantages of DNA vaccines include the low cost and ease of manufacturing of the plasmid vector and the potential ability of unmethylated CpG motifs in the bacterial plasmid vector to stimulate the innate branch of the immune system [8].

One potential target for a vaccine strategy is tyrosinase (Tyr), a prototypical melanocytic differentiation antigen that is expressed homogenously by most melanoma specimens [9-11] and which elicits spontaneous CD8^+^ T cell responses [12-14]. DNA vaccines have elicited measurable immune responses against Tyr and gp100, another melanocytic differentiation antigen, in pre-clinical mouse models [15], dogs with spontaneous melanoma [16-19], and humans [20-22]. We have shown in pre-clinical mouse models that immunization with DNA coding the xenogeneic orthologues of self-antigens is an effective strategy for inducing immunologic responses and overcoming immunologic tolerance [23-25].

Based on studies performed by our group, a xenogeneic (human) Tyr DNA vaccine was approved by the U.S. Department of Agriculture for the treatment of melanoma in dogs in 2007 [19]. Our group has also shown that Tyr-reactive CD8^+^ T cell responses could be induced in melanoma patients following administration regimens comprising intramuscular (IM) injection of DNA plasmid vectors encoding the human Tyr (pINGhuTyr) and mouse Tyr (pINGmuTyr) [20]. The rationale for incorporating a xenogeneic variant of Tyr in the immunization regimen either before or after immunization with pINGhuTyr was the potential to enhance immunogenicity due to the slight divergence of polypeptide sequences between species [15].

Accumulated clinical experience in the field of DNA based immunization indicates that an important challenge in DNA vaccine development is the identification of an effective method for delivery. While DNA immunization by conventional IM and intradermal injection have been utilized in a wide range of disease indications, concerns regarding suboptimal potency have led to the evaluation of alternative methods for delivery [26]. These methods include physical methods for enhancing intracellular DNA delivery such as particle-mediated epidermal delivery (PMED) of DNA-coated gold particles and *in vivo* electroporation (EP). EP involves injecting plasmid DNA solutions into targeted tissues, followed by electric pulses that transiently increase cell membrane permeability to facilitate intracellular uptake of the plasmids [8]. EP has been demonstrated to increase expression of the delivered genes in targeted tissues compared to conventional injection of plasmid DNA alone and causes minimal tissue damage [27]. In non-clinical [28] and clinical studies [29,30], EP mediated DNA vaccine delivery has demonstrated significant enhancement in the induction of cell mediated immune responses.

In order to assess the potential for *in vivo* EP to enhance IM delivery of the pINGmuTyr plasmid DNA vaccine, we performed a phase I trial in human melanoma patients. The objectives of the study were to characterize the safety of the administration procedure at escalating DNA dose levels as well as the resulting Tyr specific immunological response. Although the previous clinical study of DNA based Tyr immunization evaluated a combined syngeneic and xenogeneic Tyr immunization regimen, the present phase I study included only the xenogeneic pINGmuTyr construct. The rationale for this was the favorable outcome of the xenogeneic immunization in canine melanoma [16-19] and the logistical advantages of testing a single vector construct.

## Results

### Patient demographics

Of the 24 patients enrolled on this trial, 21 received all five vaccinations and were evaluable for immune responses and survival. One patient (Tyr-20) withdrew informed consent, another patient (Tyr-12) experienced progressive disease (PD) after the second vaccination and the third patient (Tyr-16) experienced syncope after the first vaccination and was removed from the trial. Among the 21 evaluable subjects, three were administered at the 0.2 mg dose level, three at the 0.5 mg dose level, and 15 at the 1.5 mg dose level.

Patient demographics are listed in Table 1. The median age of the patients was 60 and 71% were male. All patients had a Karnofsky performance status ≥ 90%. Most (67%) had resected stage III disease. All eligible stage II-III patients had refused adjuvant IFN-α therapy, after a thorough discussion with a physician investigator. One patient with recurrent stage IV disease had previously been treated with adjuvant IFN-α for resected stage IIB disease, developed recurrence while on IFN-α and had undergo resection of a solitary lung metastasis. Other adjuvant therapies included radiation (four patients, one with concurrent temozolomide) and temozolomide as single-agent (one patient) or with isolated limb infusion with dactinomycin/melphalan (one patient). One patient underwent radiofrequency ablation of a solitary liver metastasis. 18 of 24 patients enrolled in this study were HLA-A01 positive; One patient was HLA-A02 positive; Three were HLA-A2402 positive; one was HLA-A25 and two were HLA-A03 as showed in Table 2.

**Table 1:** Patient demographics

** Characteristic**	**Overall**	
	**n**	**%**
**Number**	24	
**Age**		
Range	35-70	
Median	60	
**Sex**		
Male	17	71%
Female	7	39%
**Stage**		
II	4	16.5%
III	16	67%
IV	4	16.5%
**Karnofsky performance status**		
90%	7	29%
100%	16	67%
Unknown	1	4%
**Prior therapy**		
None	17	71%
Temozolomide alone	1	4.1%
Radiation alone	2	8.3%
RSA ablation	1	4.1%
Interferon	1	4.1%
Temozolomide +	1	4.1%
Radiation		
Temozolomide +	1	4.1%
Isolated limb infusion		

**Table 2:** Immune monitoring summary and clinical outcomes of patients who produced a measureable immune response

**Dose (mg)**	**Patient**	**HLA Type**	HLA/A*0101 Tetramer ^**+**^	**CD8+ IFNγ+**	**Phenotype Tetramer**^**+ **^**CD8**^**+ **^T cells*
0.2	Tyr-1	A0101	-	-	
	Tyr-2	A1101	ND	-	
	Tyr-3	A01	ND	-	
0.5	Tyr-4	A0101	-	-	
	Tyr-5	A0101	-	-	
	Tyr-6	A2501	ND	-	
1.5	Tyr-8	A2402	ND	-	
	Tyr-9	A0101	+	-	CCR7^low^,CD45RA^low^, CD27^int^, CD28^int^
	Tyr-11	A0101	-	-	
	Tyr-12#	A02	ND	ND	
	Tyr-13	A2402	ND	-	
	Tyr-14	A0301/A2402	ND	-	
	Tyr-16#	A0101	ND	ND	
	Tyr-18	A0101	-	-	
	Tyr-19	A0101	-	-	
	Tyr-20#	A0101	ND	ND	
	Tyr-21	A03	ND	-	
	Tyr-22	A0101	-	-	
	Tyr-23	A0101	-	-	
	Tyr-24	A0101	-	+	
	Tyr-25	A0101	-	+	
	Tyr-26	A0101	+	+	CCR7^low^,CD45RA^low^, CD27^int^, CD28^low^
	Tyr-27	A0101	+	-	CCR7^low^,CD45RA^low^, CD27^int^, CD28^int^
	Tyr-28	A0101	+	-	CCR7^low^,CD45RA^low^, CD27^int^, CD28^int^

* Low = 0–30%, intermediate (int) =30–60%, and high = 60–100% of cells that are tetramer positive. # Patient Tyr-12, 16 and 20 did not finish vaccine protocol. ND = not done. “+” = Patients scored positive for tetramer-reactive CD8^+^ T cell or Tyr-reactive CD8^+^IFN-γ^+^ T cell response; “-” = Patients scored negative for tetramer-reactive CD8^+^ T cell or Tyr-reactive CD8^+^IFN-γ^+^ T cell response.

### Adverse events and survival

The use of EP in the clinical setting was pretty feasible and there were no logistic or technical issues associated with its use in this study. There was minimal toxicity noted for 24 patients. Only one patient (Tyr-16) in the 1.5 mg cohort experienced grade 3 syncope immediately following administration of the first dose, with loss of consciousness for approximately 30 seconds and diaphoresis, requiring hospitalization. This was deemed unlikely to be related to vaccine toxicity and more likely of vasovagal origin. The only other grade 1/2 toxicity noted in >20% of patients was grade 1 injection site reaction (46%). After a median period of 40.9 months, median overall survival has not yet been reached.

### Increase in tyrosinase-specific tetramer-reactive CD8^+^ T-cells after immunization

**Figure 1:**
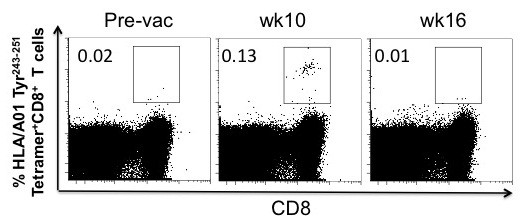
***Increase in tyrosinase***_***243-251***_HLA*A010-restricted tetramer-reactive CD8^***+***^***T cells following mouse tyrosinase DNA vaccination.*** Multiparameter flow cytometry was performed at baseline, prior to the third vaccination (week 10) and after the fifth vaccination (Week 16). Patient peripheral blood mononuclear cells (PBMCs) were cultured for 10 days with a pool of tyrosinase peptides. Representative dot plots from Patient Tyr-26 reveal an increase in the frequency of tetramer-reactive CD8^+^ T cells in this patient, with the peak at Week 10.

**Figure 2:**
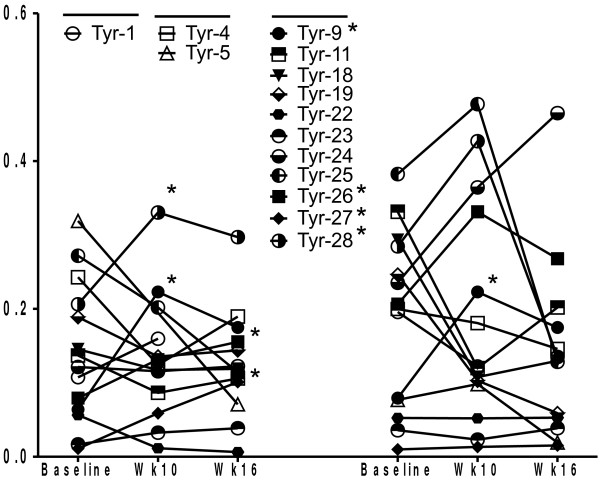
***Changes in tyrosinase***_***146-156***_***and tyrosinase***_***243-251***_HLA*A0101-restricted tetramer-reactive CD8^***+***^***T cells following mouse tyrosinase DNA vaccination.*** Each point refers to the mean of triplicate values. Most patients also underwent two peripheral blood draws at baseline one week apart prior to receiving vaccination. The values at baseline represent the mean of both of these time-points. * Refers to patients with increase in tetramer-reactive CD8^+^ cells. (Patient Tyr-9 scored positive for both Tyr tetramers, patient Tyr-26, 27 and Tyr-28 had a single Tyr tetramer positive after vaccination).

We performed multiparametric flow cytometry on patient PBMC samples at baseline and after the third dose (Week 10) and fifth dose (Week 16) using tetramers for the HLA-A*01 restricted peptides Tyr_146-156_ and Tyr_243-251_. Tetramer analysis was restricted to the 14 patients who were HLA-A*01 positive. There were not enough T cells from patient Tyr-3 for tetramer staining. Four of the 14 patients (28.6%) had detectable tetramer-reactive CD8^+^ T cells at either Weeks 10 or 16. Representative dot plots from Patient Tyr-26 are shown in Figure 1, while Figure 2 describes the change in frequency of tetramer-reactive CD8^+^ T cells for all 14 patients.

### Phenotypic analysis of the tetramer-reactive CD8^+^ T cells reflects an effector cell population

**Figure 3:**
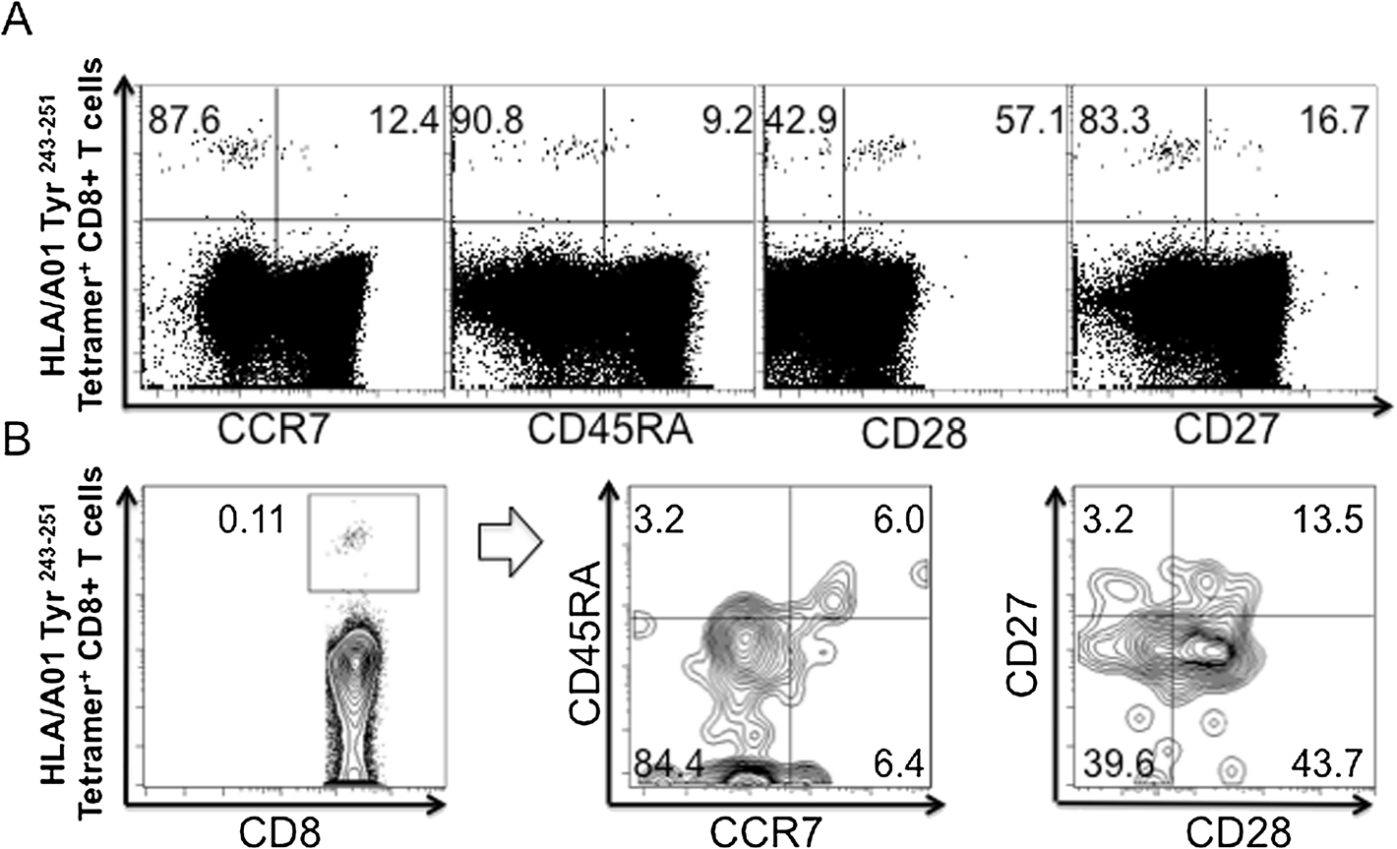
***CCR7, CD45RA, CD27 and CD28 subpopulations in tyrosinase***_***243-251***_HLA*A0101-restricted tetramer-reactive CD8^***+***^***T cells are consistent with an effector phenotype.*** PMBCs were analyzed by tetramer assay after *in vitro* culture with tyrosinase peptide pool. Representative dot plots for CD3^+^CD8^+^ T cells of patient Tyr-26 at Week 16 show the expression of CCR7, CD45RA, CD28 and CD27 on tyrosinase_243-251_ HLA*A0101 tetramer reactive CD8^+^ T cells **(A)**. Further characterization in contour plots of CD3^+^CD8^+^tetramer^+^ T cells revealed that these cells were CCR7^-^CD45RA^-^CD27^lo^CD28^mid^, consistent with an effector phenotype **(B)**.

Chemokine receptor 7 (CCR7) and CD45RA are often used to subtype the CD8^+^ T cell population, providing the following phenotypes: naïve cells (CCR7^+^CD45RA^+^), central memory cells (CCR7^+^CD45RA^-^), effector memory cells (CCR7^-^CD45RA^-^), and effector cells (CCR7^-^CD45RA^+^) [31]. Tetramer-reactive CD8^+^ T cells from all three patients were also CCR7^low^CD45RA^low^, consistent with an effector phenotype (Figure 3).

In addition, it has been proposed that CD27 and CD28 are markers that further characterize the CCR7^-^CD45RA^-^ population into four subpopulations with different effector/cytolytic function: EM1 (CD27^+^CD28^+^), EM2 (CD27^+^28^-^), EM3 (CD27^-^CD28^-^) and EM4 (CD27^-^CD28^+^) [32]. The EM1 subtype has a phenotype very similar to the CCR7^+^CD45RA^-^ central memory cells, while the EM2 and EM3 subtypes express mediators associated with effector cells. CD27 expression was found to be intermediate in the tetramer-reactive CD8^+^CCR7^low^CD45RA^low^ population of all three patients, which can be observed in a population of activated T cells that are undergoing differentiation to develop effector functionality. CD28 expression was intermediate in two specimens and low in one specimen, also consistent with the process of differentiation towards an effector phenotype.

### Increases in CD8^+^IFN-γ^+^ T cells after immunization and evidence of polyfunctionality

**Figure 4:**
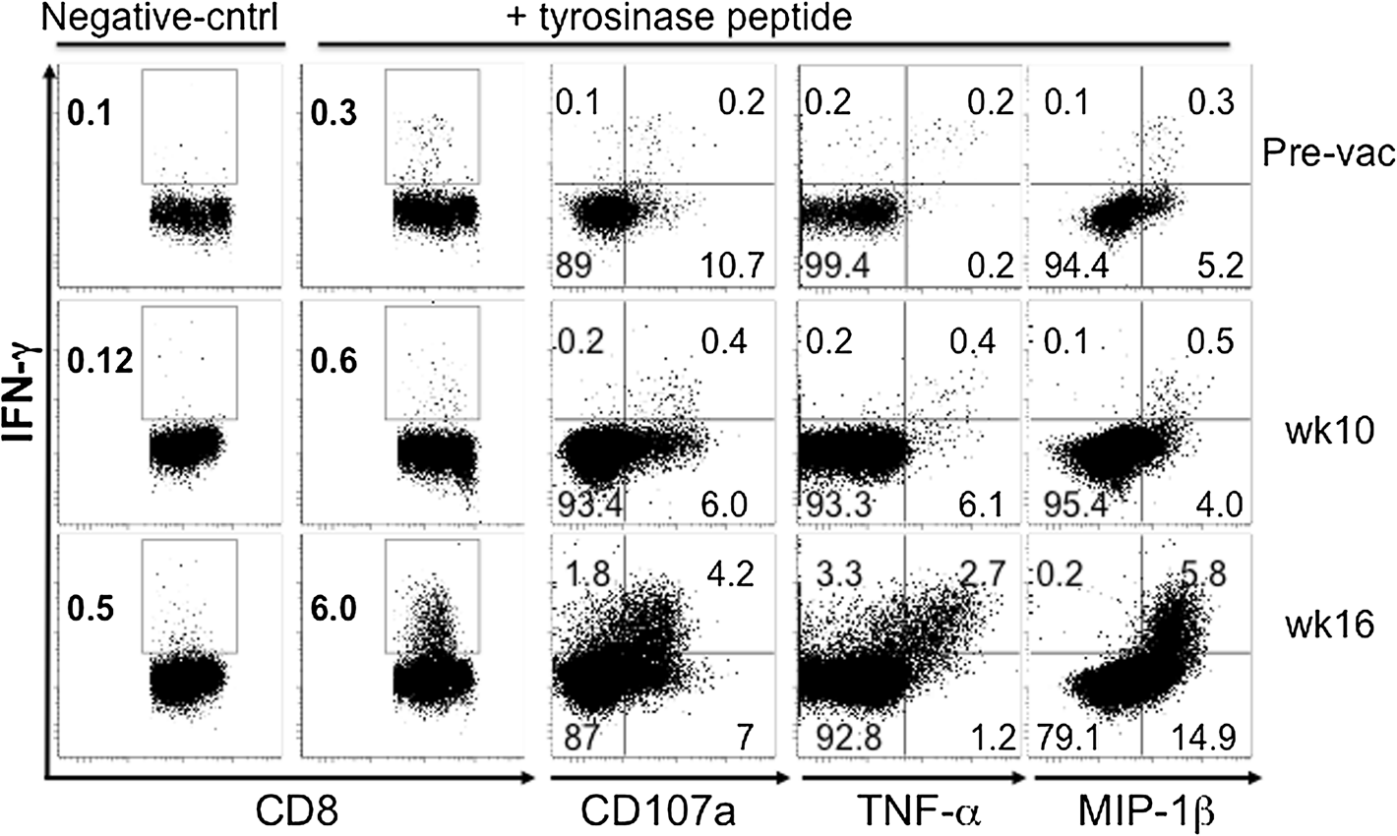
***Increase in tyrosinase-reactive CD8***^***+***^***IFN-γ***^***+***^***T cells following mouse tyrosinase DNA vaccination.*** Intracellular cytokine staining was performed by multiparameter flow cytometry after culturing patient PBMCs with a tyrosinase peptide epitope pool for 10 days. These are representative dot plots of CD3^+^CD8^+^ T cells from Patient Tyr-25 who had an increase in CD8^+^IFN-γ^+^ cells following vaccination. There was evidence of polyfunctionality, including cells that were both IFN-γ^+^, and either CD107a^+^, MIP-1β^+^ or TNF-α^+^.

We next performed ICS to enumerate the intracellular cytokine profile of CD8^+^ T cells in all 21 patients. Three patients were found to have an increase in Tyr-reactive CD8^+^IFN-γ^+^ T cells following immunization. Representative dot plots from Patient Tyr-25 are shown in Figure 4. There was evidence of an increase not only in Tyr-reactive CD8^+^ T cells that expressed IFN-γ but also evidence of polyfunctional cytokine responses, consisting of combinations of IFN-γ and MIP-1β, TNF-α and CD107a. Immunization elicited both an increase in tetramer-reactive CD8^+^ T cells as well as a cytokine response in one of the three patients (Patient Tyr-26).

**Figure 5:**
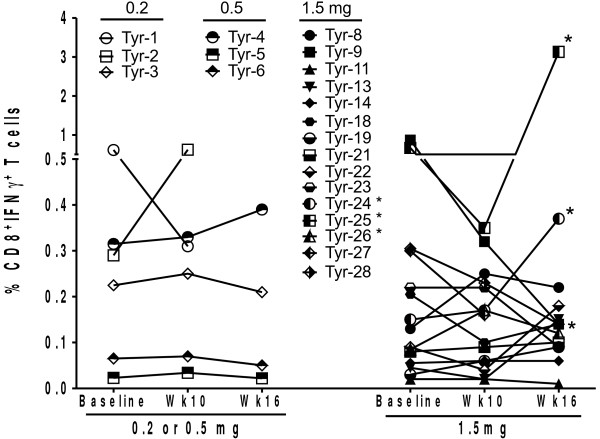
***Changes in frequency of tyrosinase-reactive CD8***^***+***^***IFN-γ***^***+***^***T cells following mouse tyrosinase DNA vaccination.*** Each point refers to the mean of triplicate values in intracellular cytokine staining assays. Most patients also underwent two peripheral blood draws at baseline one week apart prior to receiving vaccination. The values at baseline represent the mean of both of these time-points. * Refers to patients with increase in CD8^+^ IFN-γ + T cells.

Overall, six of the 15 patients at 1.5 mg dose level experienced a Tyr-reactive CD8^+^ T-cell response after stimulation with the Tyr peptide epitope pool as detected by tetramer or ICS analysis. No Tyr-reactive CD8^+^ T cell response was detected in patients at 0.2 mg and 0.5 mg dose cohort. In the responding patients, the increase from baseline to peak response ranged from 1.5-fold to 2.8-fold in the tetramer assay and from 2.1-fold to 3.3-fold in the ICS assay. Positive responses were seen as early as at Week 10 and up to Week 16. All six patients with a CD8^+^ T cell response had received the 1.5 mg dose. These data are summarized in Figure 5 and Table 2.

### CTLA-4 blockade recalled Tyr-reactive CD8^+^ T cell responses in a patient

**Figure 6:**
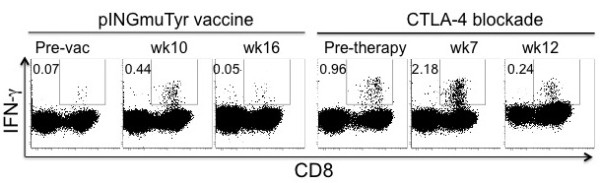
***CTLA-4 blockade induced tyrosinase-specific CD8***^***+***^***IFN-γ***^***+***^***T cell responses.*** ICS was performed on PBMCs from Patient Tyr-25 at various time-points. He developed a tyrosinase-specific CD8^+^IFN-γ^+^ T cell response following vaccination. Subsequently, he developed recurrent disease and was treated with ipilimumab. A persistent tyrosinase-specific CD8^+^IFN-γ^+^ response was noted prior to ipilimumab therapy, which increased with ipilimumab therapy and peaked after the second dose (at Week 7).

Patient Tyr-25 was a 62-year-old man with a history of multiple primary melanomas. He underwent a left axillary lymph node dissection in October 2007 for recurrent melanoma, followed by adjuvant chemoradiation with temozolomide. He was then enrolled on the DNA vaccine trial in April 2009 but developed progressive disease after completing the trial. In October 2009, he initiated therapy with ipilimumab 10 mg/kg every three weeks for four doses. Because the patient had developed a Tyr-reactive CD8^+^ T cell response following vaccination, we quantified changes in this response after he was treated with ipilimumab. A significant increase in Tyr-reactive CD8^+^IFN-γ^+^ T cells was detected after the second ipilimumab dose (Figure 6). Unfortunately, he had rapidly progressive disease and died in April 2010.

### Robust NY-ESO-1 specific CD4^+^ and CD8^+^ T cell response during DNA vaccination

**Figure 7:**
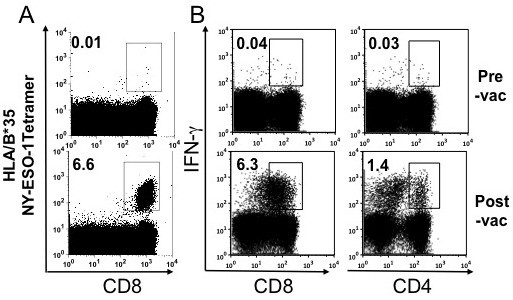
***Mouse tyrosinase DNA vaccination and subsequent NY-ESO-1 specific tetramer and IFN-γ response.*** Patient Tyr-2 was known to have baseline seropositivity for NY-ESO-1. Thawed PBMCs from pre- and post- pINGmuTyr vaccination were cultured for 10 days with NY-ESO-1 overlapping peptides before tetramer analysis and ICS. Following vaccination, there was an increase in **(A)** HLA/B*35 NY-ESO-1_94-102_ tetramer-reactive CD8^+^ T cells and **(B)** NY-ESO-1 specific CD4^+^ and CD8^+^ IFN-γ^+^ T cells.

Patient Tyr-2 was a 70-year-old woman with a history of a resected melanoma of unknown primary site arising in her left inguinal area. After receiving five doses of the pINGmuTyr vaccine at the 0.2 mg dose level, she developed recurrent disease with vitiligo even though a Tyr-reactive CD8^+^IFN-γ^+^ T-cell response was not detected after stimulation with the peptide epitope pool. She was known to have pre-existing baseline antibody titers against NY-ESO-1, a cancer-testis antigen. As such, we characterized changes in NY-ESO-1 specific immune responses. Following vaccination, she developed a robust NY-ESO-1 specific CD8+ T-cell response in terms of both HLA/B*35 NY-ESO-1_94-102_ tetramer-reactive cells (Figure 7A) and IFN-γ^+^ cells (Figure 7B). This patient also had a rapid expansion of NY-ESO-1 specific CD4^+^IFN-γ^+^ T cells after vaccination.

## Discussion

In this phase I study, we demonstrated that a mouse Tyr DNA vaccine could be safely administered via *in vivo* EP to melanoma patients. The only common toxicity was grade 1 injection site reaction in 46% of patients. Six of 15 patients (40%) in the 1.5 mg dose cohort developed Tyr-reactive CD8^+^ T cell responses following stimulation, defined by a ≥3 standard deviation increase in baseline reactivity by tetramer or ICS assays. We didn’t detect Tyr-reactive CD8^+^ T cell response in 0.2 mg and 0.5 mg dose cohort patients. 4 HLA-A*01 positive experienced an increase in the frequency of Tyr-specific tetramer-reactive CD8^+^ T cells following vaccination. Three also had an increase in Tyr-reactive CD8^+^ IFN-γ^+^ T cells; one patient had an increase in both tetramer-reactive and CD8^+^ IFN-γ^+^ T cells. 6 of 21 (28.6%) patients had an increase either in Tyr-reactive tetramer-reactive and/or CD8^+^IFN-γ^+^ T cells following vaccination. The fact that all six melanoma patients with a detectable Tyr-reactive T cell response received the 1.5 mg dose provides preliminary indications of a potential dose response. Results from other clinical testing suggest that increases in DNA dose may offer an avenue to further enhance the magnitude and breath of immune response [29]. However, it is also important to note that, due to the limited number of epitopes represented within the peptide pool and absence of HLA-A*24 tetramer reagents, the immunologic responses detected may underestimate the overall number of patients developing increased anti-Tyr immune reactivity.

Although differences in study design and patient population enrolled in our pINGmuTyr studies conducted to date precludes direct comparative analysis, the results obtained in the present study from analysis of a population of HLA-A*01 and HLA-A*24 positive patients are qualitatively similar to our previous study, where the pINGmuTyr and pINGhuTyr DNA vectors were administered via the IM route to HLA-A*02 positive patients. In that study, 7 of 18 (39%) HLA-A*02 positive patients experienced an increase either in Tyr-reactive tetramer or CD8^+^IFN-γ^+^ T cells following vaccination [20]. However, in the current study, only tetramer responses to two HLA-A*01 epitopes could be examined in 14 of the 21 evaluable patients, potentially underestimating responses to other unassessed epitopes presented by other HLA types. Moreover, it is unclear what effect the monovalent immunization against muTyr alone performed in the present study may have had on the magnitude and frequency of response, especially with the presence of point mismatches between the human and murine sequences in one of the two HLA-A*01 epitopes and the single HLA-A*24 epitope included in the peptide pool used for immunogenicity analysis.

We have also previously used polyfunctional cytokine responses – combinations of IFN-γ with MIP-1β, TNF-α and CD107a – to quantify the “quality” of the immune response [33,34]. While polyfunctional CD8^+^ T cell responses were seen in this trial, they were not as qualitatively robust as in two of our previous DNA vaccination trials with IM injection of a mouse and human gp100 DNA vaccine or with granulocyte-macrophage colony-stimulating factor DNA and a tyrosinase and gp100 peptide vaccine [35]. The results of the current trial were comparable to a recent trial of a gp100 DNA vaccine via PMED [22].

Our group has previously reported that ipilimumab therapy can augment humoral and cellular immune responses against different melanoma-associated tumor antigens, including gp100 and NY-ESO-1, a prototypical cancer-testis antigen, whose expression is restricted to the testes and certain malignant tissues, including melanoma [34,36]. Here, we observed a similar phenomenon, where a Tyr-reactive CD8^+^IFN-γ^+^ T cell response induced by vaccination was subsequently enhanced by the administration of two doses of ipilimumab six months later for recurrent disease. NY-ESO-1 is relatively immunogenic and baseline immune responses have been reported in melanoma patients. It has previously been noted by our group and others that humoral and T-cell mediated responses to NY-ESO-1 are highly correlated [37]. As such, we were interested in studying the impact, if any, of the pINGmuTyr vaccine on NY-ESO-1 specific CD8^+^ T cell responses in a patient with baseline NY-ESO-1 seropositivity. Although a Tyr-specific immune response could not be demonstrated following stimulation with the limited peptide epitope pool used in our assays, this patient was the only person in the study to develop vitiligo. To our surprise, she developed a robust NY-ESO-1 specific CD4+ and CD8^+^ T-cell response by tetramer staining and ICS following vaccination. Such an observation of “epitope spreading” suggests that vaccination could potentially induce immune responses not only against the target protein but also potentially against other immunogenic components of the malignant cell. This process may occur when vaccination generates a cytotoxic immune response that leads to apoptosis of the malignant cell and the release of cellular contents that may then be processed by APCs to generate additional immune responses against other targets. Vaccination may also generate an inflammatory milieu at sites of disease, providing costimulatory signals necessary for the recruitment and activation of antigen-specific effector cells.

Unfortunately, the augmentation of Tyr-reactive immune responses by ipilimumab and the induction of NY-ESO-1 specific responses by the pINGmuTyr vaccine respectively in the patients above did not translate into clinical benefit. The former developed recurrent disease five months after vaccination and died within six months of starting ipilimumab; the second patient developed recurrence after the completion of five vaccinations. Numerous possibilities may explain this discrepancy and highlight the challenges of successful vaccine development, including the downregulation of cancer-specific antigens to permit malignant cells to escape immune detection and destruction or the elicitation of diverse mechanisms, e.g. regulatory T cells that suppress the immune system [38].

The relative inefficacy of a monovalent vaccine – at least one based on gp100 – is suggested by the phase III trial of ipilimumab, in which patients received ipilimumab or a gp100 peptide vaccine or the combination. Both ipilimumab arms (alone or with gp100 vaccine) had superior overall survival compared to the vaccine-only arm. However, there was no difference between the ipilimumab arms, suggesting minimal benefit from the vaccine. Hence, this seminal phase III trial represents a significant advancement in the treatment of refractory melanoma, is an important triumph in the field of immunotherapy but also highlights the significant challenges to the successful development of a melanoma vaccine.

## Conclusions

To date, our group has evaluated peptide and DNA vaccines against gp100 and/or tyrosinase delivered via the IM, PMED or EP route. Within the limitation of the heterogeneous study designs (i.e. variation in HLA types enrolled, testing of regimens combining syngeneic and xenogeneic antigens vs. xenogeneic alone), existing immunogenicity assays (possible underestimation of immune response based on the scope of the peptide pool used for assessment) and small patient numbers involved, all of these approaches appear to produce antigen-specific immune responses and none appears clearly superior to the others. It is interesting to note the dose-dependent immune response in this study. As we and others survey the field, possible future trials may involve further optimization of the vaccine and a multivalent vaccine against such targets as Tyr, gp100 and NY-ESO-1. Targeting multiple antigens expressed on melanoma cells may create a more immunogenic vaccine that also decreases the ability of the malignant cells to escape immune detection and destruction. In addition, the combination of such a multivalent vaccine with ipilimumab remains an attractive proposition. While the phase III trial above involved the simultaneous administration of vaccine and ipilimumab, the anecdotal observation here and elsewhere by our group suggests a sequential administration of vaccine followed by ipilimumab may potentiate and expand the immune responses produced by vaccination [36].

## Methods

### Eligibility criteria

All patients were diagnosed with stage IIB-IV malignant melanoma, histologically confirmed at the Memorial Sloan-Kettering Cancer Center (MSKCC). Patients had undergone prior surgical resection of primary or recurrent disease, were disease-free and had either declined adjuvant IFN-α therapy or had previously developed recurrence while on it. Additional eligibility criteria included a Karnofsky performance status ≥ 80%, HLA-A1, -A2, -A24 or -B35 positivity, the absence of detectable brain metastases and adequate organ and bone marrow function. Exclusion criteria included prior chemotherapy, vaccination using tyrosinase DNA sequence, protein or peptides, systemic immunosuppressive therapy, surgery or radiotherapy within four weeks of study entry, active autoimmune disease other than vitiligo, patient with a history of syncope and/or seizures, and women who were pregnant or < 3 months post partum or nursing. The study (NCT00471133) was reviewed and approved by the MSKCC Institutional Review Board and all patients provided informed consent.

### Study design and treatment plan

Cohorts of three patients were sequentially accrued to three dose levels of the mouse tyrosinase pINGmuTyr plasmid construct by *in vivo* EP. 0.2, 0.5 or 1.5 mg of plasmid DNA was injected every three weeks for a total of five doses. Treatment was discontinued upon the development either of dose-limiting toxicity (DLT) or of recurrent disease requiring systemic treatment or radiation therapy. DLT was defined as any grade ≥3 toxicity or grade ≥ 2 allergic/immunologic toxicity, as per the Common Terminology Criteria for Adverse Events, version 3.0. With no DLT observed at any of the tested dose levels, study enrollment was expanded at the 1.5 mg plasmid DNA dose level using the same administration regimen until a total of 15 subjects evaluable for immunogenicity were enrolled at that dose level.

### DNA vaccine construct

Mouse Tyr plasmid DNA was previously sequenced and introduced into the pING vector by our group [39]. The pINGmuTyr construct has been extensively tested in both pre-clinical studies and clinical trials [17,20]. This vector is in accordance with the FDA’s Points to Consider for DNA vaccination. Clinical-grade pINGmuTyr DNA was prepared by the MSKCC GMP compliant Gene Transfer Core Facility and tested for endotoxin, sterility and animal safety. The vaccine was administered IM at a tissue site with intact lymphatic drainage. It was administrated as a single injection for each time point. No injection was given at a location in which the draining lymph nodes had been removed. The site of immunization was alternated for each of the immunizations (e.g. left deltoid for the first, third and fifth and right deltoid for the second and fourth). The vaccine was administered using an EP system appropriate for IM DNA delivery in the clinical setting provided by Ichor Medical Systems, Inc. (San Diego, CA), according to the manufacturer’s instructions. Immediately after the injection, a series of brief electrical pulses was applied to the local tissue. The stimulation lasted for about 0.5 seconds and resulted in localized contractions of the muscles at the injection site.

### Evaluations at baseline and during therapy

At baseline, a complete history and physical examination was performed, along with a baseline ophthalmologic examination to exclude pre-existing retinal or choroidal disease. Routine blood work, chest imaging (X-ray or CT) and a brain MRI were also obtained. Patients with a history of resected metastatic disease also underwent appropriate radiographic imaging to ensure they were disease-free at time of study entry.

For immune function monitoring, blood samples were drawn one week before and immediately prior to the first vaccination and at Weeks 10 and 16, which corresponds to after the third and fifth doses respectively. To ensure the acquisition of a sufficient quantity of peripheral blood mononuclear cells (PBMC), leukopheresis was performed at baseline and Week 16, if possible. In addition, patients underwent clinical and radiologic monitoring as indicated to assess for disease recurrence.

### Immune function monitoring

Tetramer and intracellular cytokine staining (ICS) assays were performed using multiparametric flow cytometry, as previously described [33,40]. For this initial analysis, we selected eight well characterized CD8^+^ T cell epitopes of human tyrosinase based upon the previous published literature, and validated the pool of these peptides for in vitro T cell culture. Briefly, thawed PBMCs were incubated at a 1:1 ratio with irradiated autologous PBMCs pulsed with the following peptide pool at 10 μg/ml each: HLA-A1-restricted TYR_146-156_ (SSDYVIPIGTY) and TYR_243-251_ (KCDICTDEY); HLA-A2-restricted TYR_1-9_ (MLLAVLYCL), TYR_8-17_ (CLLWSFQTSA) and TYR_369-377_ (YMDGTMSQV); HLA-A24 restricted TYR_206-214_ (AFLPWHRLF); HLA-B35 restricted TYR_309-320_ (TPRLPSSADVEF) and; HLA-B44 restricted TYR_192-200_ (SEIWRDIDF) (JPT Peptide Technologies, Berlin, Germany).

Cells were harvested at Day 10 and analyzed immediately by tetramer staining. For ICS, cells were additionally incubated for 20 minutes with PE-Cy5-CD107a (5 μl/ml; BD Pharmingen) prior to re-stimulation with the preceding tyrosinase peptide pool for 2 hours. Five μg/ml each of Brefeldin A and monensin (BD Biosciences, San Jose, CA) were then added for another 4 hours. The following tetramers and fluorochrome-labeled antibodies were used: HLA-A*0101-restricted TYR_146-156_ (SSDYVIPIGTY) and TYR_243-251_ (KCDICTDEY) tetramer (Tetramer Core, Lausanne Branch, Ludwig Institute of Cancer Research, Lausanne, Switzerland), PE-Cy7-CD3, APC-CD27, PerCPCy5.5-CD28, APC-Interleukin (IL)-2, PE-Macrophage inflammatory protein (MIP)-1β, and FITC-Interferon (IFN)-γ (BD Pharmingen, San Jose, CA), Pacific blue-CD3, APC-AF750-CD8, PE-Cy7-Tumor necrosis factor (TNF)-α (eBioscience, San Diego, CA), ECD-CD4, ECD-CD45RA (Beckman Coulter Inc., Fullerton, CA) and FITC-CCR7 (R&D Systems, Minneapolis, MN). Cells were analyzed using a CYAN-ADP flow cytometer with Summit software (Dako Cytomation California Inc., Carpinteria, CA). Analysis was performed using FlowJo software (version 9.1; TreeStar, Inc., Ashland, OR).

### Statistical analysis

A T cell response at any post-vaccination time-point was considered positive if it was ≥3 standard deviations greater than the mean value at baseline and had an absolute value >0.1%. Differences between groups were analyzed using Fisher’s exact test. Progression-free survival (PFS) and overall survival were estimated using the Kaplan-Meier method.

## Abbreviations

CTL: Cytotoxic T lymphocyte; PBMC: Peripheral blood mononuclear cell; ICS: Intracellular cytokine staining; (TNF)-α: Tumor necrosis factor-α; HLA: Human leukocyte antigen; CCR7: Chemokine receptor 7; CTLA-4: Cytotoxic T-lymphocyte antigen-4; IM: Intramuscular; EP: Electroporation; DLT: Dose-limiting toxicity; PD: Progressive disease; OS: Overall survival.

## Competing interests

The authors declare that they have no competing interests.

## Authors’ contributions

JY, ANH, and JDW designed research. JY, GYK, MA, ZM, ST, DH, PC, GS, RC, and KSP performed research. JY, GYK, KSP and JDW analyzed data; JY, GYK, DH and JDW wrote paper. All authors read and approved the final manuscript.
